# Excision of a Solitary Fibrous Tumor in the Sciatic Notch with Sciatic Nerve Compression – A Rare Clinical Case

**DOI:** 10.1055/s-0042-1757302

**Published:** 2023-07-31

**Authors:** Tiago Fontainhas, Ana Sofia Costa, Rui Sousa, Ana Flávia Resende, João Nelas, David Pereira

**Affiliations:** 1 Departamento de Ortopedia e Traumatologia, Centro Hospitalar Tondela-Viseu, Viseu, Portugal

**Keywords:** sciatic nerve, soft tissue neoplasms, solitary fibrous tumor, pleural

## Abstract

We present the clinical case of a 41-year-old woman with no relevant personal history. The patient complained of diffuse self-limiting abdominal pain, and we incidentally detected an extra-abdominal, extraperitoneal tumor mass at the level of the right sciatic notch. The abdominal complaints were gone during the initial follow-up, but the patient developed sciatica radiating to the right foot and electric shock-like pain. A computed tomography (CT)-guided biopsy revealed a low-grade mesenchymal neoplasm of the soft tissues with characteristics consistent with a solitary extrapleural fibrous tumor. The pelvis team of the orthopedics department received the patient for surgical excision of the lesion. The procedure occurred with no complications, and we excised the totality of the lesion with tumor-free margins. An anatomopathological examination was compatible with the biopsy assessment. The excision of the lesion resulted in complete resolution of the sciatic nerve compression-related symptoms.

## Introduction

Solitary fibrous tumors (SFTs) are rare, slow-growing neoplasms of mesenchymal origin that account for < 2% of all soft tissue tumors. Solitary fibrous tumors can appear virtually anywhere in the body, although they are more frequent in an intrathoracic location. 1 Extrapleural SFTs are more common at the intra-abdominal level and may be intraperitoneal, retroperitoneal, or pelvic. 2 From a clinical point of view, these tumors are usually asymptomatic until they are large enough to cause compressive symptoms. These dimensions vary considerably depending on the tumor mass location, ranging from 1 to 40 cm. 1 2 3 The present article presents a case report of a patient with an SFT in the sciatic notch, a rare location, with symptoms of sciatic nerve compression.

## Case Report

**Fig. 1:**
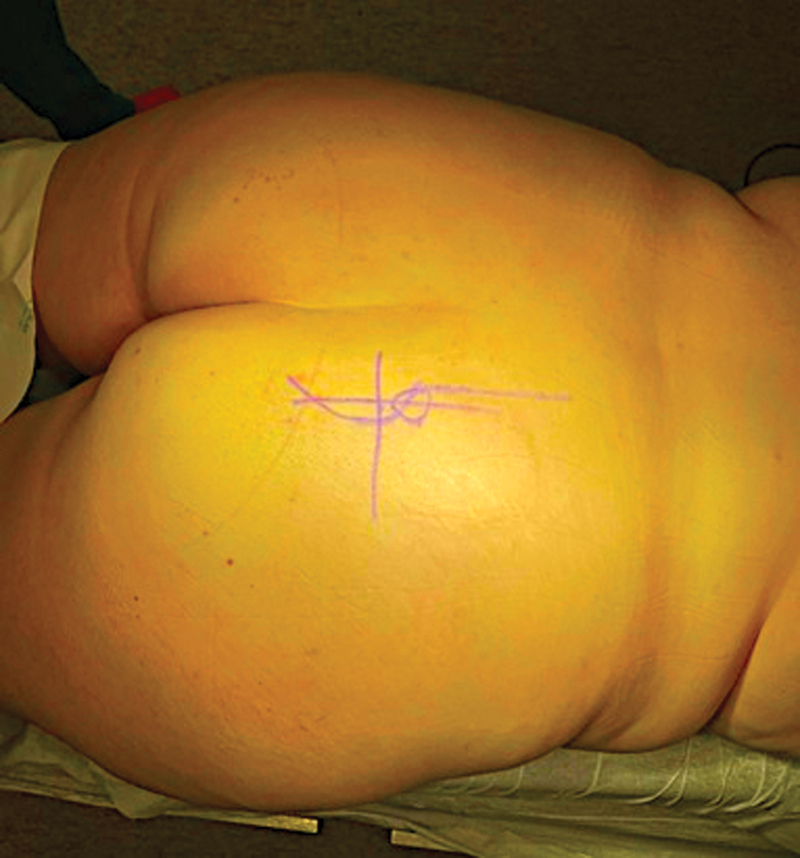
Sciatic notch, tumor location, and incision line determination with the support of fluoroscopy and based on an abdominopelvic computed tomography scan.

**Fig. 2:**
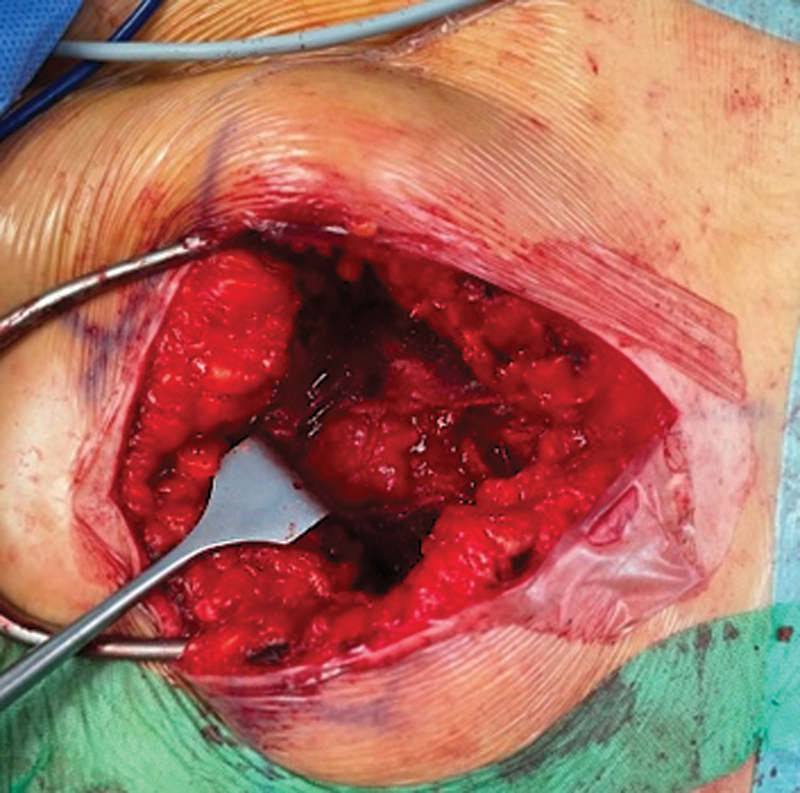
From a posterior approach to the sciatic notch, we identified the piriformis muscle, located the tumor mass, and excised it.

**Fig. 3:**
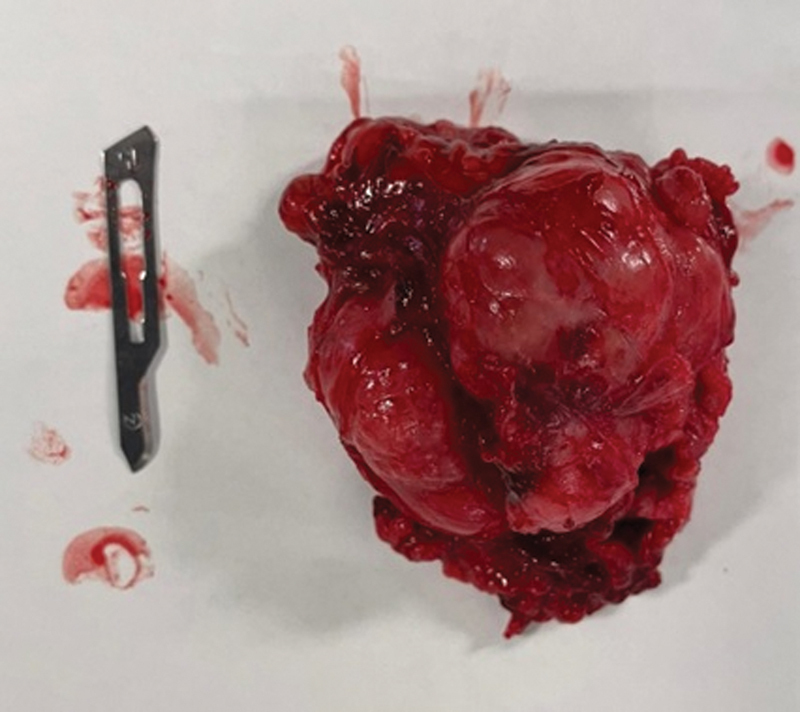
Excised tumor.

The pelvis team of the orthopedics department received a 41-year-old woman with no relevant history due to an incidental finding on a pelvic computed tomography (CT) scan performed due to abdominal pain. The abdominal complaints were gone during the initial follow-up, but the patient developed sciatic-like pain radiating to the right lower limb. The pain was neuropathic (similar to an electric shock) and did not respond to medication. Additional diagnostic tests showed an extra-abdominal, extraperitoneal tumor mass located in the right sciatic notch and potentially compressing the sciatic nerve. A CT-guided biopsy described the lesion as an extrapleural SFT. We proposed the surgical excision of the tumor lesion. The complete removal of a solid mass occurred with no complications ( Figs. 1 , 2 , and 3 ). An anatomopathological examination was consistent with the previously established diagnosis and confirmed the tumor-free margins. Surgery resulted in the complete resolution of the symptoms of sciatic nerve compression, and the patient was discharged from the orthopedics department.

## Discussion

Solitary fibrous tumors are rare neoplastic lesions. They are often asymptomatic, and their diagnosis is usually incidental. Their variable location also translates into different sizes at diagnosis, depending on the mass effect required to cause symptoms. 1 A contrast CT scan usually demonstrates a well-delimited, hypervascularized, and often lobulated tumor with necrotic areas. 4 Ideally, one must request a biopsy to diagnose and classify the malignancy because there are numerous differential diagnoses. 1 Since this type of tumor is rare, there are no guidelines based on randomized clinical trials. As a result, a multidisciplinary approach similar to that used to treat soft tissue sarcomas is acceptable. After the diagnosis, the consensual treatment of an STF is surgical excision of the lesion with tumor-free margins. 1 2 3 4 The high variability of lesion locations requires surgical planning on a case-by-case basis. For this patient, the surgical team opted for a posterior approach to the sciatic notch. Using a CT scan as a reference, we marked the incision site with fluoroscopy support ( Fig. 1 ). Following a modified posterior approach to the right sacroiliac joint (most distal incision), we identified the piriformis muscle ( Fig. 2 ). The tumor mass was immediately adjacent to this muscle, and it was easily palpable. The excision occurred with no complication ( Fig. 3 ), and the anatomopathological examination confirmed the presence of tumor-free margins. This type of tumor is frequently benign, but it may be aggressive at the local level. However, some SFTs are malignant, and it is difficult to predict this behavior. 5 6 Obtaining tumor-free margins during surgical excision is critical to prevent recurrence and improve prognosis. A small series of long-term case studies have demonstrated local recurrence rates of 8%, but actual values are likely to be higher. 4 7 Yet, the response of malignant tumors is much less durable, with recurrence rates of up to 63% despite complete lesion resection. 4 Several variables tried to identify a malignant behavior ( Table 1 ). 1 Still, the overall prognosis of SFTs is significantly superior compared with those of other soft tissue neoplasms, with 5- and 10-year survival rates of 59 to 100% and of 40 to 89%, respectively. 4 8 One of the largest studies on SFTs reports survival rates of 89% at 5 years and of 73% at 10 years. 3 This is why we recommend follow-up after an SFT resection even though guidelines are lacking. The presence of malignancy features warrants a tighter follow-up ( Table 1 ). 1

**Table 1:** Tumor characteristics associated with malignant behavior

Study	Tumor characteristics
Gold et al. 2	Recurrent tumor
	Gross or microscopic positive margins after tumor excision
	Size > 10 cm
	> 4 mitoses/10 high-power field
	Increased nuclear pleomorphism
	Increased cellularity
	Presence of malignant components
Demicco et al. 3	Age > 55 years old
	Size > 15 cm
	≥ 4 mitoses/10 high-power field
	Tumoral necrosis

Solitary fibrous tumors are rare and poorly studied neoplasms. Although often asymptomatic and diagnosed incidentally, they may cause symptoms related to mass effects. In addition, their presentation is highly variable because they can affect virtually any area of the human body. Surgical excision is the consensual treatment, and long-term follow-up is critical.
