# Supplementary material for: Excision of a Solitary Fibrous Tumor in the Sciatic Notch with Sciatic Nerve Compression – A Rare Clinical Case
Source: Rev Bras Ortop (Sao Paulo). 2023 Jul 31;59(Suppl 1):e98–e100. doi: 10.1055/s-0042-1757302 (PMC11254444; doi:10.1055/s-0042-1757302)
Supplement: Related texts

# Research article: Excisão de tumor fibroso solitário na chanfradura ciática com compressão do nervo ciático – Um raro caso clínico

**Keywords:** neoplasias de tecidos moles, nervo ciático, tumor fibroso solitário pleural

## Abstract

É apresentado um caso clínico de uma paciente de 41 anos, sem antecedentes pessoais de relevo, que foi estudada por dor abdominal difusa autolimitada, tendo sido detectada incidentalmente uma massa tumoral extrabdominal, extraperitoneal ao nível da chanfradura ciática direita. Durante o estudo do caso, a doente resolveu as queixas abdominais, mas desenvolveu quadro de ciatalgia, irradiada para o pé direito e dor tipo choque elétrico. Biópsia guiada por TAC revelou tratar-se de uma neoplasia mesenquimatosa dos tecidos moles, de baixo grau, com características que a enquadram em um tumor fibroso solitário extrapleural. A paciente foi referenciada para a equipe de bacia do serviço de ortopedia para excisão cirúrgica da lesão, tendo sido operada sem intercorrências – excisão total da lesão, com margens livres, foi obtida e o exame anatomopatológico foi compatível com o realizado na biópsia. Após excisão da lesão, ocorreu resolução completa dos sintomas relacionados com compressão do nervo ciático.

## Introdução

Os tumores fibrosos solitários (TFSs) são tumores raros de crescimento lento e de origem mesenquimatosa que correspondem a < 2% de todos os tumores de tecidos moles. Os TFSs podem surgir em praticamente todo o corpo, embora sejam mais frequentes em localização intratorácica. 1 Tumores fibrosos solitários em localização extrapleural são mais comuns a nível intra-abdominal, podendo ser intraperitoneais, retroperitoneais ou pélvicos. 2 Do ponto de vista clínico, estes tumores são normalmente assintomáticos até atingirem tamanho suficiente para originar sintomas compressivos – as dimensões são extremamente variáveis dependendo da localização da massa tumoral, podendo medir entre 1 e 40 cm. 1 2 3 No presente artigo, é apresentado um relato de caso de uma paciente com um TFS em localização rara, a chanfradura ciática, com sintomatologia de compressão do nervo ciático.

## Relato de Caso

Paciente do sexo feminino, 41 anos, sem antecedentes de relevo, referenciada para a equipe de bacia do departamento de ortopedia por um achado incidental em TAC pélvica, realizada para estudo de dor abdominal. Durante o acompanhamento inicial, a doente resolveu as queixas abdominais, mas desenvolveu dor tipo ciática, com irradiação para o membro inferior direito, com qualidade neuropática (dor tipo choque elétrico) e que não resolvia com medicação. Meios complementares de diagnóstico caracterizam a presença de uma massa tumoral extra-abdominal, extraperitoneal, localizada na chanfradura ciática direita, com provável compressão do nervo ciático. Biópsia guiada por TAC descreve a lesão como um tumor fibroso solitário extrapleural. A paciente foi proposta para excisão da lesão tumoral, tendo sido operada sem intercorrências – com remoção completa de uma massa sólida ( Figs. 1 , 2 e 3 ). O exame anatomopatológico foi consistente com o diagnóstico previamente estabelecido e confirmou margens livres. Após a cirurgia, houve resolução completa dos sintomas de compressão do nervo ciático e a paciente recebeu alta clínica do departamento da ortopedia.

**Fig. 1:** Com apoio de fluoroscopia, e tendo por base a TAC abdominopélvica, foi marcada a chanfradura do ciático, a localização do tumor e a linha de incisão.

**Fig. 2:** Foi realizada uma abordagem posterior da chanfradura ciática. Uma vez identificado o músculo piriforme, identificou-se a massa tumoral e efetuou-se a sua excisão.

**Fig. 3:** Tumor excisado.

## Discussão

Os TFSs são lesões tumorais raras. Dado que muitas vezes são assintomáticos, são comumente diagnosticados de forma acidental. A localização variável também se traduz em tamanhos diversos no momento do diagnóstico, em função do efeito de massa necessário para causar sintomatologia. 1 TAC contrastada habitualmente demonstra um tumor bem delimitado, hipervascularizado e frequentemente lobulado, com áreas de necrose. 4 Idealmente, uma biópsia é realizada com o intuito de diagnosticar e classificar a malignidade da lesão, uma vez que os diagnósticos diferenciais são inúmeros. 1 Dada a raridade deste tipo de tumor, não existem diretrizes baseadas em ensaios randomizados clínicos. Como resultado, atualmente é aceita uma abordagem multidisciplinar semelhante à realizada para o tratamento de sarcomas de tecidos moles. Uma vez diagnosticado o TFS, o tratamento consensual é exérese cirúrgica da lesão com margens livres. 1 2 3 4 O planeamento cirúrgico tem de ser feito caso a caso, dada a elevada variabilidade de localizações das lesões. Neste caso clínico, a equipe de cirurgiões optou por uma abordagem posterior da chanfradura ciática. Utilizando a TAC como referência, foi marcado o local da incisão com apoio de fluoroscopia ( Fig. 1 ). Seguindo uma abordagem posterior modificada da articulação sacroilíaca direita (incisão mais distal), o músculo piriforme foi identificado ( Fig. 2 ) e a massa tumoral foi encontrada imediatamente adjacente, sendo facilmente palpável. A excisão foi realizada sem intercorrências ( Fig. 3 ), confirmando-se a presença de margens livres no exame anatomopatológico. O comportamento deste tipo de tumor é geralmente benigno, mas potencialmente agressivo a nível local. Contudo, uma fração destes pacientes tem TFS com comportamento maligno – sendo este desenvolvimento difícil de prever. 5 6 A obtenção de margens livres na excisão cirúrgica é importante para prevenir recorrência e melhorar o prognóstico. Uma pequena série de casos estudos a longo prazo demonstrou níveis de recorrência local de 8%, mas os níveis reais de recorrência provavelmente são mais elevados. 4 7 Em tumores com comportamento maligno, contudo, a resposta é muito menos duradoura, com recorrência em até 63% casos apesar de uma resseção completa da lesão. 4 Várias variáveis foram descritas para tentar identificar o comportamento maligno tumoral ( Tabela 1 ). 1 Ainda assim, o prognóstico geral dos TFSs é significativamente superior ao de muitas outras neoplasias de tecidos moles, com taxa de sobrevivência de 59 a 100% aos 5 anos, e de 40 a 89% aos 10 anos. 4 8 Um dos maiores estudos sobre os TFS reporta taxas de sobrevivência de 89% aos 5 anos e de 73% aos 10 anos. 3 Por este motivo, o acompanhamento está recomendado após a resseção de TFSs, apesar de não haver diretrizes estabelecidas. A presença de características de malignidade dita um acompanhamento mais apertado ( Tabela 1 ). 1

**Tabela 1:** Características tumorais associadas com comportamento maligno

Estudo	Características do tumor
Gold et al. 2	Tumor recorrente
	Margens macro ou microscopicamente positivas do tumor excisado
	Tamanho > 10 cm
	> 4 mitoses/10 campos de grande ampliação (HPF)*
	Pleomorfismo nuclear aumentado
	Celularidade aumentada
	Presença de componentes malignos
Demicco et al. 3	Idade > 55 anos
	Tamanho > 15 cm
	≥ 4 mitoses/10 campos de grande ampliação (HPF)*
	Necrose tumoral

Abreviação: HPF, high-power field.

Os TFSs são neoplasias raras e pouco estudadas. Apesar de frequentemente assintomáticos e diagnosticados acidentalmente, por vezes provocam sintomatologia relacionada com efeito de massa, sendo a apresentação extremamente variável – dada a possibilidade de se poder manifestar em qualquer área do corpo humano. É consensual a exérese cirúrgica como tratamento, sendo importante o acompanhamento a longo prazo.
